# Association of sperm DNA fragmentation with abstinence duration, seminal oxidative stress and age: a call to consider abstinence in andrological diagnostics

**DOI:** 10.3389/fruro.2026.1926391

**Published:** 2026-09-22

**Authors:** Ralf Henkel, Mohamed Arafa, Ahmad Majzoub, Haitham El Bardisi, Mausumi Das

**Affiliations:** 1 LogixX Fertility, Theale, Berkshire, United Kingdom; 2 Department of Medical Bioscience, University of the Western Cape, Bellville, South Africa; 3 Department of Urology, Hamad Medical Corporation, Doha, Qatar; 4 Department of Urology, Weill Cornell Medicine-Qatar, Doha, Qatar; 5 Department of Andrology, Cairo University, Cairo, Egypt; 6 College of Medicine, Qatar University, Doha, Qatar; 7 Glasgow Royal Infirmary & University of Glasgow, Glasgow, United Kingdom; 8 Chelsea and Westminster Hospital, Assisted Conception Unit, Imperial College, London, United Kingdom

**Keywords:** diagnostic test, ejaculatory abstinence, semen analysis, seminal oxidative stress, sperm DNA fragmentation

## Abstract

**Introduction:**

According to World Health Organization (WHO) recommendations, the abstinence period for a semen analysis is 2 to 7 days. Other professional medical societies recommend shorter abstinence periods. Since sperm DNA fragmentation (SDF) is positively associated with oxidative stress, age and abstinence, this study aimed at investigating a threshold of abstinence for the diagnostic use of SDF testing.

**Methods:**

In this retrospective study, SDF (HaloSperm) and oxidative stress (MiOXSYS) were tested in 2782 consecutive patients, attending the Department of Urology at Hamad Medical Corporation in Doha, Qatar. Patients were stratified according to the abstinence.

**Results:**

Results showed significant trends of higher SDF and oxidative stress with longer periods of abstinence. ROC curve analysis with 20% SDF as classification variable resulted in thresholds of >4 days for abstinence, >37 years for age and 59.9 mV for oxidation-reduction potential. Multiple linear regression analysis revealed abstinence, age and oxidative stress as strong independent predictors of high SDF.

**Discussion:**

When requesting and interpreting an SDF test, abstinence, age and oxidative stress have to be considered. A maximum of 4 days of abstinence is recommended.

## Introduction

According to the definition of the World Health Organization (WHO), infertility is a disease where a couple does not achieve pregnancy after at least 12 months of regular unprotected sexual intercourse (1). Globally, an estimated one in six people suffer from infertility at some stage in their lifetime, with approximately half of the cases caused or contributed to by the male partner. Since this disease has significant adverse effects on general health, mental and psychological well-being, as well as finances and the relationship of affected couples (2–5), even leading to social stigma, depression, domestic violence and divorce (6, 7), infertility must be recognised as a globally under-recognised public health problem (8).

In the andrological workup, the semen analysis according to WHO guidelines (9) is the primary diagnostic tool for identifying male factor infertility. Apart from semen volume and viscosity, this widely standardized analysis examines basic semen parameters such as sperm concentration, motility, normal sperm morphology and sperm viability. Today, despite its limitations and repeated criticism (10–13), the standard semen analysis is still the cornerstone of andrological fertility diagnosis (14).

As part of an extended and advanced examination, the “WHO laboratory manual for the examination and processing of human semen” suggests testing for sperm DNA fragmentation (SDF) and seminal oxidative stress, respectively, as they have emerged as clinical biomarkers of male infertility. Oxidative stress and elevated SDF are intricately linked and have been associated with poor reproductive outcomes after different methods of assisted reproduction, including pregnancy loss (15–18).

However, despite all the standardization, for the period of sexual abstinence, which has been shown to influence semen quality and consequently clinical decision-making as well as reproductive outcomes (19–23), a range of between 2 and 7 days of ejaculatory abstinence is recommended. The American Urological Association (AUA), American Society of Reproductive Medicine (ASRM) and the European Association of Urology (EAU) also follow this recommendation by the WHO. On the other hand, the Nordic Association for Andrology (NAFA) and the European Society of Human Reproduction and Embryology (ESHRE) Special Interest of Andrology Group, recommend a narrower range of 3 to 4 days of ejaculatory abstinence (24).

Although numerous articles investigating the effects of ejaculatory abstinence on semen parameters have been published thus far (21, 25), insufficient information is available about the ideal period of sexual abstinence for normal SDF. SDF is positively associated with seminal oxidative stress, age and the duration of sexual abstinence (26–28) and may therefore be influenced by these parameters. Consequently, positive or negative outcomes of SDF testing may affect the clinical management of patients. Therefore, this study aims to investigate an ideal period of abstinence for normal SDF, considering oxidative stress and the patients’ age as confounding factors.

## Materials and methods

### Patients

In this retrospective cohort study, semen analyses, seminal oxidative stress and sperm DNA fragmentation of a total of 2782 consecutive patients, aged between 18 and 73 years, attending the infertility unit at the Department of Urology at Hamad Medical Corporation in Doha, Qatar, for fertility evaluation, between January 2016 and January 2018 were analysed. Otherwise, no additional inclusion or exclusion criteria were used.

The study was approved by the Ethics Committee of the Medical Research Center of Hamad Medical Corporation. A waiver of informed consent was obtained for this study.

### Semen analysis

Semen samples were collected in sterile plastic containers after times of sexual abstinence between 1 and 10 days. After liquefaction of the samples for 30 minutes at 37 °C, a standard semen analysis was performed according to the fifth Edition of the WHO Laboratory Manual for the Examination and Processing of Human Semen (29).

### Determination of sperm DNA fragmentation

SDF was determined by using the Halosperm G2 test (Halotech DNA SL, Madrid, Spain) according to the manufacturer’s instruction. In brief, a semen sample was diluted with phosphate-buffered saline to adjust the sperm concentration to a maximum of 20×10^6^ sperm/mL. Thereafter, 50 µL of this suspension were mixed with low-melting-point agarose and 8 µL of the suspension placed on a slide and covered with a coverslip. After the agarose-sperm suspension was solidified, the coverslip was removed and the sample denatured, lysed and stained. Subsequently, excess stain was washed off and the slide evaluated in a brightfield microscope. Sperm with intact DNA produce a dispersion halo while those with fragmented DNA do not produce such a halo or only a much smaller halo of a size up to one third of the core diameter (30). Since an SDF level of >20% is generally accepted as high, we also used this threshold (31–33).

### Determination of seminal oxidative stress

For the measurement of the seminal OS, the MiOXSYS^®^ system (Aytu BioScience, Englewood, CO) was used. In brief, 30 µL of liquefied semen were placed on the sample port of the sensor. The static oxidation-reduction potential (sORP) was displayed after approximately 4 minutes. For the analyses in this study, the actual ORP readings in milli Volts (mV) and the normalized values (normalized ORP) expressed as mV/10^6^ sperm/mL were recorded (34).

### Statistical analysis

For the statistical analyses, MedCalc Statistical Software (v 23.5.5; MedCalc Software Ltd., Ostend, Belgium) was used. After testing for normal distribution with the Shapiro-Wilk test, data was expressed as mean ± SD and median with interquartile range (IQR). Non-parametric tests (Spearman rank correlation and Mann-Whitney test), Chi-squared test, and Kruskal-Wallis test with subsequent Jonckheere-Terpstra trend test were conducted. Patients were stratified as into 4 groups of sexual abstinence (1 to 2 days, 3 to 4 days, 5 to 6 days, and 7 or more days) according to Ayad et al. (35). ROC curve analyses for abstinence, age, sORP, sperm concentration and the total number of DNA-damaged sperm were performed with an SDF threshold of >20% as classification criterion. For the investigation of the effects of causative/contributing factors (age, sexual abstinence and oxidative stress) to SDF, odds ratios (OR) were calculated using the Cochrane-Mantel-Haenszel test and multiple linear regression analysis with variables entered into the model using the backward method.

Since not all parameters were investigated for all (n=2782) patients, lower numbers were recorded for ejaculate volume, SDF, total number of DNA-damaged sperm, morphology, sORP, normalized ORP, total and progressive motility, and vitality.

A P-value of P<0.05 was considered significant.

## Results

**Table 1** shows the patients’ summary statistics of this study while **Table 2** provides results of Spearman Rank correlations between the various parameters taken in this study. While normal sperm morphology showed no association with any of the investigated parameters, all other variables were associated. Specifically, the duration of ejaculatory abstinence showed significant positive correlations with sperm concentration, ejaculate volume, SDF, the total number of DNA-damaged sperm, sORP, and total motility. SDF and sORP were significantly associated with all parameters except normal sperm morphology and ejaculate volume, and age and morphology, respectively.

**Table 1:** Summary statistics.

	n	Minimum	Maximum	Mean	Median	SD	IQR
Abstinence (d)	2782	1.00	10.00	3.88	3.00	1.63	3.00 to 5.00
Age (yrs)	2782	18.00	73.00	35.85	35.00	7.75	30.00 to 40.00
Conc. (10^6/^ml)	2782	0.60	160.00	32.56	28.00	25.54	12.00 to 47.00
Ejaculate volume (mL)	2781	0.50	27.00	3.00	2.50	1.73	2.00 to 4.00
SDF (%)	762	5.00	100.00	29.22	25.00	19.06	15.00 to 37.00
Total Number of DNA-damaged Sperm (10^6^)	761	0.578	378.00	28.69	15.84	38.37	7.33 to 35.00
Total Number of Motile DNA-damaged Sperm (10^6^)	760	0.00	151.20	12.94	7.49	16.41	3.51 to 15.08
Normal Morphology (%)	791	0.00	100.00	4.81	4.00	7.31	2.00 to 5.00
Normalized ORP (mV/10^6^ sperm/mL)	2781	-6.33	222.07	5.17	1.88	10.46	1.00 to 4.38
Progr. Motility (%)	2781	0.00	50.00	11.44	10.00	11.51	0.00 to 20.00
sORP (mV)	2781	-65.30	666.20	56.74	50.90	37.37	37.38 to 68.73
Total Motility (%)	2770	0.00	100.00	47.51	50.00	18.66	40.00 to 60.00
Vitality (%)	604	0.00	96.00	46.08	48.00	19.37	32.00 to 60.00

C, Combined data for abstinence new (updated 23.03.26) n=2783.mc1.

**Table 2:** Spearman Rank correlations between abstinence, age, sperm concentration, SDF, total number of DNA-damaged sperm, total and progressive motility, sORP and normalized ORP, normal sperm morphology, ejaculate volume, and sperm viability.

		Abstinence (d)	Age (yrs)	Conc. (10^6/^ml)	Ejaculate volume (ml)	SDF (%)	Total number of DNA-damaged sperm (10^6^)	Normal sperm morphology (%)	sORP (mV)	Normalized ORP (mV/10^6^ sperm/mL)	Total motility (%)	Progr. motility (%)
Age (yrs)	r	0.024	1									
	P	0.2138										
	n	2782										
Conc. (10^6/^ml)	r	0.100	0.001	1								
	P	<0.0001	0.9738									
	n	2782	2782									
Ejaculate Volume (ml)	r	0.168	-0.098	-0.047	1							
	P	<0.0001	<0.0001	0.0124								
	n	2781	2781	2781								
SDF (%)	r	0.124	0.178	-0.112	0.060	1						
	P	0.0006	<0.0001	0.0020	0.0992							
	n	762	762	762	761							
Total Number of DNA-damaged Sperm (10^6^)	r	0.244	0.008	0.606	0.494	0.494	1					
	P	<0.0001	0.8175	<0.0001	<0.0001	<0.0001						
	n	761	761	761	761	761						
Normal sperm morphology (%)	r	-0.017	-0.037	0.001	-0.005	-0.006	0.037	1				
	P	0.6422	0.2998	0.9668	0.8994	0.9255	0.5946					
	n	791	791	791	791	213	213					
sORP (mV)	r	0.113	0.031	0.075	0.063	0.181	0.168	0.000	1			
	P	<0.0001	0.1027	0.0001	0.0009	<0.0001	<0.0001	0.9916				
	n	2781	2781	2781	2780	762	761	791				
Normalized ORP (mV/10^6^ sperm/mL)	r	-0.026	0.013	-0.837	0.076	0.219	-0.405	-0.006	0.409	1		
	P	0.1692	0.5054	<0.0001	0.0001	<0.0001	<0.0001	0.8565	<0.0001			
	n	2781	2781	2781	2780	762	761	791	2781			
Total Motility (%)	r	-0.076	-0.123	0.341	0.033	-0.522	-0.150	0.005	-0.124	-0.378	1	
	P	0.0001	<0.0001	<0.0001	0.0830	<0.0001	<0.0001	0.8808	<0.0001	<0.0001		
	n	2770	2770	2770	2769	753	752	787	2769	2769		
Progr. Motility (%)	r	-0.020	-0.095	0.446	0.031	-0.408	-0.030	-0.031	-0.097	-0.447	0.749	1
	P	0.2957	<0.0001	<0.0001	0.0981	<0.0001	0.4087	0.3825	<0.0001	<0.0001	<0.0001	
	n	2781	2781	2781	2780	761	760	791	2780	2780	2770	
Viability (%)	r	-0.074	-0.158	0.138	0.011	-0.723	-0.348	0.063	-0.112	-0.166	0.636	0.174
	P n	0.0700	0.0001	0.0007	0.7837	<0.0001	<0.0001	0.4795	0.0060	<0.0001	<0.0001	<0.0001
		604	604	604	604	141	141	128	604	604	602	603

**Tables 3** and **4** provide results after stratification for normal (≤20%)/high (>20%) SDF (**Table 3**) and duration of sexual abstinence (**Table 4**). With regard to ejaculatory abstinence, a significantly longer period of abstinence was observed for patients with high (>20%) SDF values as compared to those patients with normal SDF values (**Table 3**). Similarly, significantly higher values were observed for age, sORP, normalized ORP, and the total number of DNA-damaged sperm. Sperm concentration, total and progressive motility as well as viability were significantly lower in patients with high SDF than in those with normal SDF values. For the ejaculatory volume and normal sperm morphology, no difference could be noticed. Stratification of ejaculatory abstinence into 4 groups (1 to 2 days, 3 to 4 days, 5 to 6 days, and 7 or more days) showed significant trends toward higher values after longer periods of abstinence for sperm concentration, ejaculate volume, SDF, sORP, and the total number of DNA-damaged sperm (**Table 4**; **Figure 1**). Interestingly, while a highly significant positive trend was found for sORP, this was not the case for normalized ORP. For total motility and viability, the trends were negative, whereas no significant effects were observed for age, normal sperm morphology, normalized ORP, and progressive motility (**Table 4**).

**Table 3:** Comparison of duration of abstinence, age, semen variables, sperm DNA fragmentation (SDF) and ORP (sORP and normalized ORP) between patients with normal (≤20%) and high (>20%) SDF.

	Categorisation for sperm DNA fragmentation		
	≤20%	>20%	P ^#^
Abstinence (d)	3.63 ± 1.48 n = 322	4.08 ± 1.68 n = 440	0.0002
Age (yrs)	34.45 ± 6.57 n = 322	37.13 ± 8.40 n = 440	<0.0001
Sperm concentration (10^6/^ml)	34.72 ± 22.57 n = 322	31.97 ± 43.23 n = 440	0.0091
Ejaculate volume (mL)	3.00 ± 2.03 n = 322	3.13 ± 1.73 n = 439	0.2072
SDF (%)	14.26 ± 3.93 n = 322	40.18 ± 18.28 n = 440	<0.0001
Total Number of DNA-damaged Sperm (10^6^)	14.50 ± 16.78 n = 322	39.10 ± 45.73 n = 439	<0.0001
Normal Morphology (%)	4.61 ± 5.26 n = 109	4.17 ± 3.84 n = 104	0.5197
Normalized ORP (mV/10^6^ sperm/mL)	2.55 ± 3.46 n = 322	3.47 ± 4.23 n = 440	<0.0001
Progr. Motility (%)	15.89 ± 11.95 n = 322	8.37 ± 10.82 n = 439	<0.0001
sORP (mV)	51.41 ± 24.17 n = 322	61.06 ± 40.19 n = 440	0.0018
Total Motility (%)	57.69 ± 14.05 n = 316	43.57 ± 19.09 n = 437	<0.0001
Sperm viability (%)	65.71 ± 16.08 n = 21	44.29 ± 18.53 n = 120	<0.0001

Means ± SD are provided.

Data are: Mean ± Standard deviation.

^#^Mann-Whitney test.

**Table 4:** Comparison of duration of abstinence, age, semen variables, sperm DNA fragmentation (SDF) and ORP (sORP and normalized ORP) between different categories of sexual abstinence.

Days of abstinence	1 to 2 days	3 to 4 days	5 to 6 days	≥ 7 days	P ^#^	Trend P ‡
Abstinence (d)	1.71 ± 0.46 n = 252 (a)(b)(c)	3.21 ± 0.41 n = 1833 (a)(d)	5.17 ± 0.38 n = 231 (b)	7.02 ± 0.21 n = 466 (c)(d)	–	–
Age (yrs)	34.92 ± 8.03 n = 252 (a)	35.83 ± 7.54 n = 1833	36.85 ± 7.79 n = 231 (a)	35.92 ± 8.34 n = 466	0.0176	0.0750
Sperm concentration (10^6^/ml)	29.90 ± 23.33 n = 252 (a)	31.10 ± 25.04 n = 1833 (b)	34.37 ± 26.13 n = 231	38.84 ± 27.32 n = 466 (a)(b)	<0.0001	<0.0001
Ejaculate volume (mL)	2.24 ± 1.31 n = 252 (a)(b)(c)	2.98 ± 1.61 n = 1832 (a)(d)	3.19 ± 1.53 n = 231 (b)	3.43 ± 2.28 n = 252 (c)(d)	<0.0001	<0.0001
SDF (%)	27.77 ± 21.09 n = 60 (a)	27.82 ± 17.98 n = 517 (b)	32.45 ± 21.78 n = 60	34.18 ± 20.23 n = 125 (a)(b)	0.0010	0.0001
Total Number of DNA-damaged Sperm (10^6^)	18.74 ± 25.61 n = 60 (a)(b)	23.93 ± 28.83 n = 516 (c)	33.16 ± 29.17 n = 60 (a)	50.96 ± 64.83 n = 125 (b)(c)	<0.0001	<0.0001
Normal Morphology (%)	5.42 ± 7.81 n = 86	4.64 ± 6.18 n = 502	3.86 ± 2.90 n = 58	5.44 ± 10.94 n = 145	0.8328	0.5526
Normalized ORP (mV/10^6^ sperm/mL)	4.39 ± 7.45 n = 252	5.30 ± 10.88 n = 1833	5.53 ± 11.35 n = 230	4.91 ± 9.66 n = 466	0.2820	0.2152
Progr. Motility (%)	11.84 ± 11.23 n = 252	11.45 ± 11.53 n = 1832	11.58 ± 11.79 n = 231	11.09 ± 11.48 n = 466	0.7893	0.3974
sORP (mV)	52.55 ± 36.29 n = 252 (a)	55.30 ± 37.93 n = 1833 (b)	54.07 ± 26.83 n = 230	66.02 ± 38.82 n = 466 (a)(b)	<0.0001	<0.0001
Total motility (%)	50.11 ± 18.26 n = 251 (a)	47.75 ± 18.69 n = 1824 (b)	46.38 ± 18.07 n = 230	45.70 ± 19.10 n = 465 (a)(b)	0.0012	<0.0001
Sperm viability (%)	49.38 ± 22.03 n = 47 (a)	46.91 ± 18.93 n = 402 (b)	47.75 ± 19.15 n = 55	40.25 ± 19.10 n = 100 (a)(b)	0.0059	0.0042

Means ± SD are provided.

Data are: Mean ± Standard deviation.

# Kruskal-Wallis test.

‡ Jonckheere-Terpstra test.

(a) groups with the same letter differ significantly in the post-hoc test according to Dunn.

**Figure 1:**
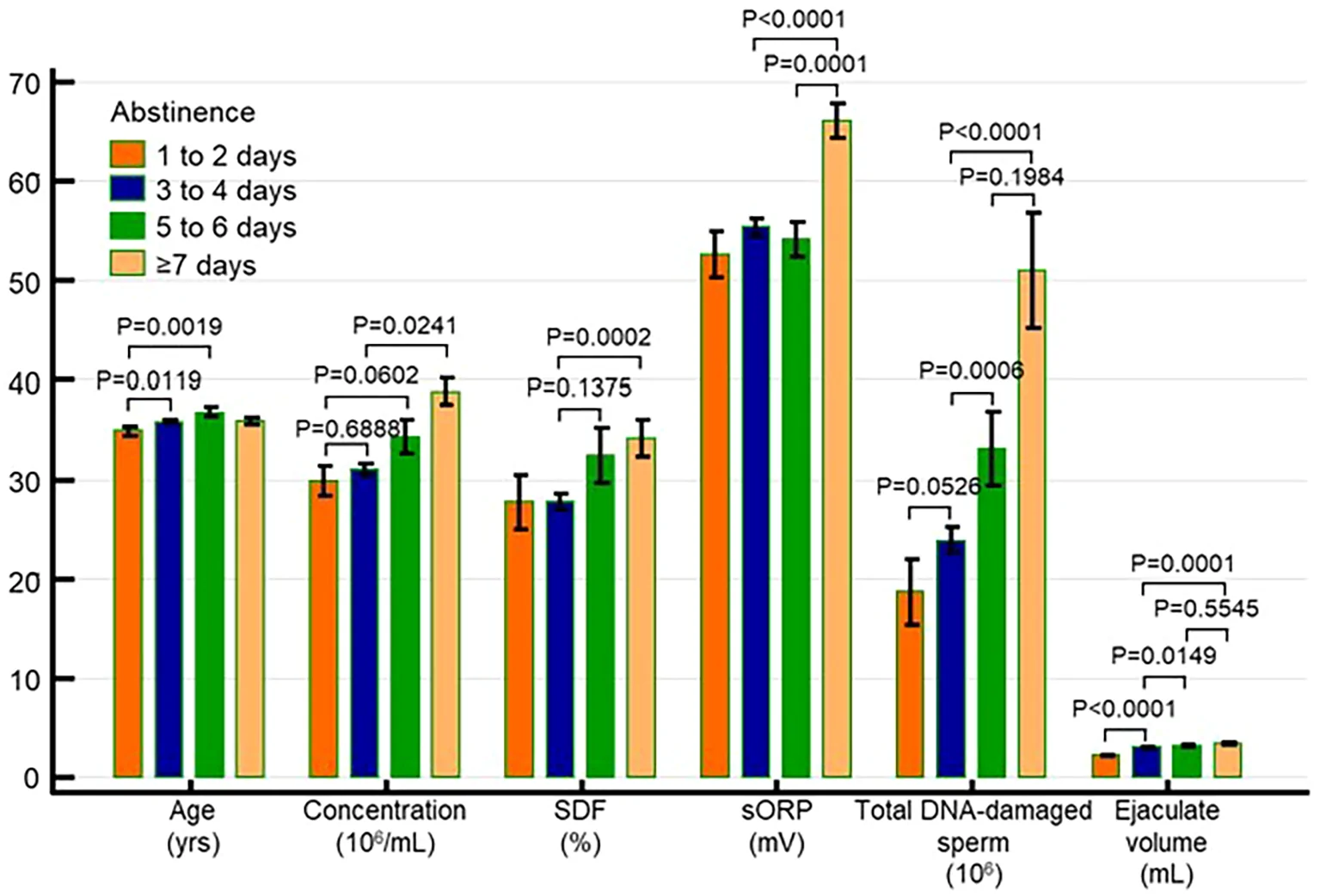
Average age (years), sperm concentration (10^6^/mL), SDF (%), sORP (mv), total number of DNA-damaged sperm (10^6^), and ejaculate volume (mL) of patients stratified for the duration of abstinence. n=2782.

ROC curve analyses to identify thresholds for sexual abstinence, age, sORP, sperm concentration, and the total number of DNA-damaged sperm using the 20% cut-off value for high SDF as a classification variable were performed (**Figure 2**). The calculation of a threshold value for sexual abstinence was significant (P = 0.0002) (AUC: 0.572; 95%CI: 0.536-0.607) for a threshold of 4 days with a sensitivity and specificity of 29.09% and 82.30%, respectively. For age and sORP similar AUCs were obtained (**Table 5**).

**Figure 2:**
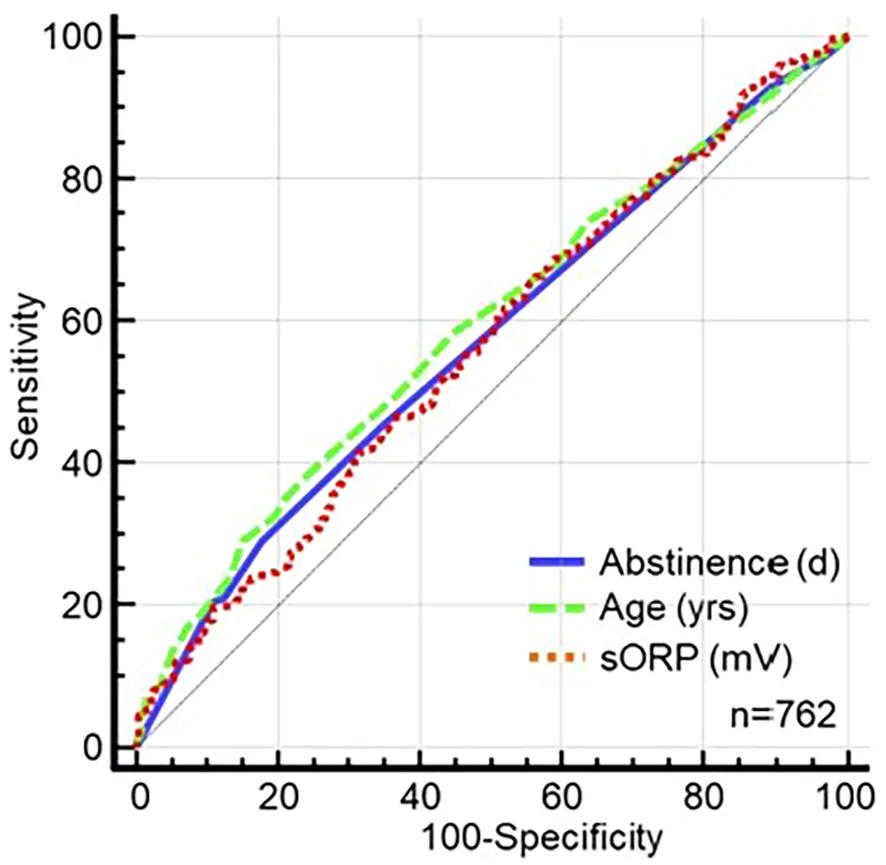
Comparison of the ROC curves for the predictive power of the duration of sexual abstinence, age, and sORP for high SDF (>20%). Sample size: n=762.

**Table 5:** ROC curve analyses for the predictive power of the duration of sexual abstinence, age, and sORP for norm/high SDF (>20%).

	AUC	95%CI	Cut-off	Sensitivity	Specificity	P-value
Abstinence (d)	0.572	0.536-0.607	>4 d	29.09%	82.30%	0.0002
Age (yrs)	0.591	0.555-0.626	>37 yrs	38.41%	75.78%	<0.0001
sORP (mV)	0.566	0.530-0.602	>59.9 mV	41.59%	68.63%	0.0016

Sample size: n=762.

Based on these cut-off values, odds ratio calculations for high SDF (>20%) revealed significantly higher probabilities for patients having elevated SDF values if the patients were sexually abstinent for more than 4 days (OR = 1.9073, P = 0.0003), had elevated sORP values (OR = 1.6640, P = 0.0008) or were older than 37 years (OR = 1.7357, P = 0.0004). When advanced age (>37 years) and higher seminal oxidative stress (>59.9 mV) as contributing factors to elevated SDF were taken in consideration, the Cochrane-Mantel-Haenszel test revealed an overall approximately two-fold higher risk of having an SDF level higher than 20% (age >37 years: OR = 2.0182; P = 0.0002). Patients with high sORP as risk factor had an overall increased odds ratio of 1.4405 (sORP >59.9 mV: P = 0.0186). Were these two confounding factors combined, the probability of high SDF values was almost three-times higher (OR = 2.8100, P<0.0001) than for young patients with normal sORP.

While the prevalence of patient presenting with an SDF level >20% after a sexual abstinence of more than 7 days was significantly (P = 0.0005) higher than after 3 to 4 days, the prevalence of high SDF after 5 to 6 days of abstinence is markedly, but not significantly (P = 0.2315) higher than after 3 to 4 days of ejaculatory abstinence (**Figure 3A**). For oxidative stress measured as sORP, the prevalence of elevated oxidative stress levels was significantly (P = 0.0017) higher after 7 or more days of abstinence as compared to 5 to 6 days (**Figure 3B**). For shorter abstinences, the differences were highly significant (P<0.0001). In contrast, no differences between the prevalence of high normalized ORP were found between the different abstinence groups (**Figure 3C**). The percentage of patients older than 37 years increased continuously from an ejaculatory abstinence of 1 to 2 days to an abstinence of 5 to 6 days. For the abstinence group of 7 or more days, however, the percentage was slightly, but not significantly (P = 0.4846), lower than for an abstinence of 5 to 6 days (**Figure 3D**).

**Figure 3:**
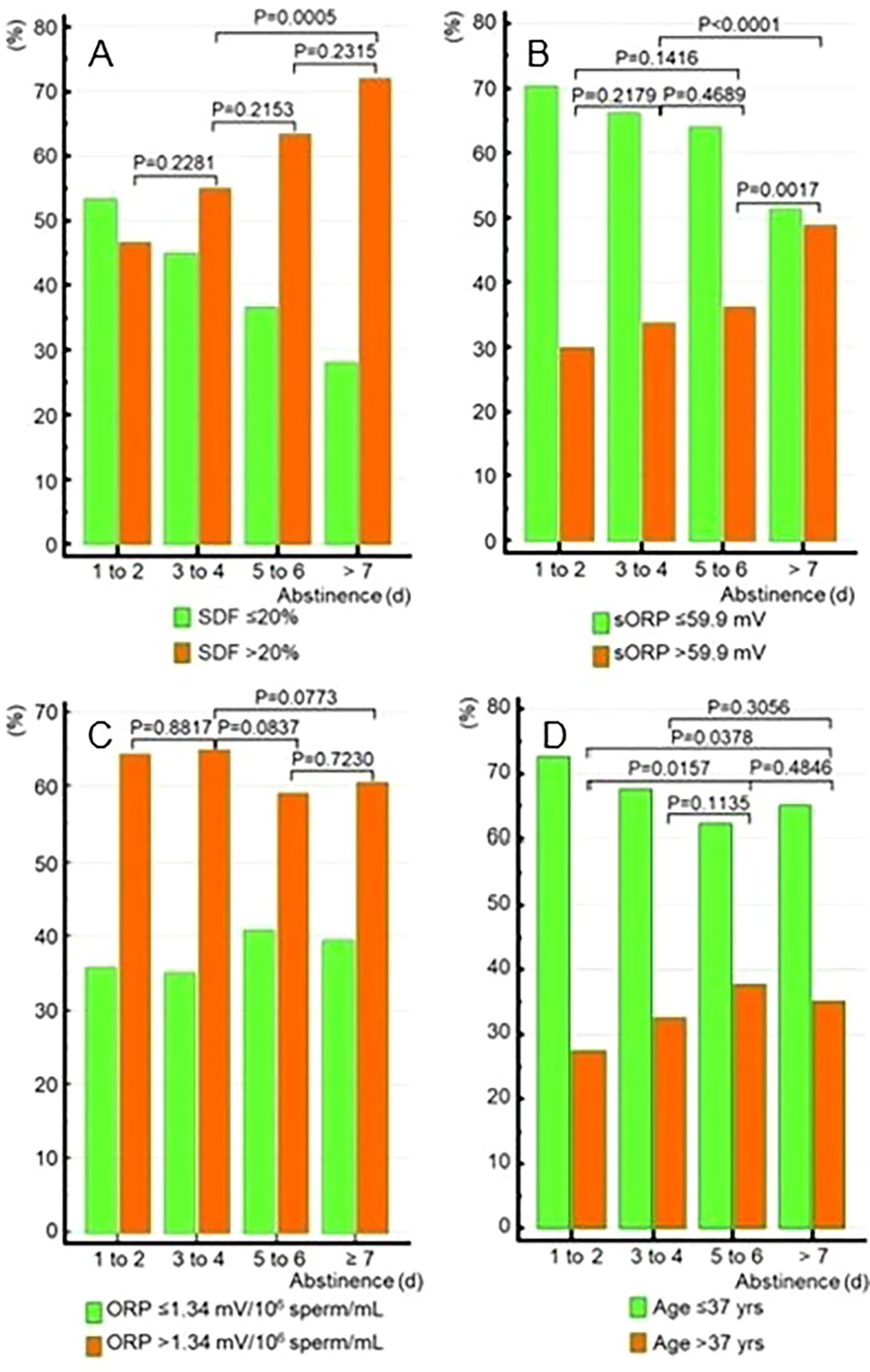
**(A)** Prevalence of high (>20%) and normal SDF values depending on the duration of sexual abstinence. (n=762). **(B)** Prevalence of high (>1.34 mV/10^6^ sperm/mL) and normal (≤1.34 mV/10^6^ sperm/mL) normalized ORP depending on the duration of sexual abstinence. (n=2781). **(C)** Prevalence of high (>59.9 mV) and normal (≤59.9 mV) sORP depending on the duration of sexual abstinence. (n=2781). **(D)** Prevalence of patients >37 years and ≤37 years depending on the duration of sexual abstinence. (n=2781).

Multiple linear regression analysis for high/low SDF as categorical outcome variable with the duration of abstinence, patient’s age and sORP as independent variables that can contribute to sperm DNA fragmentation revealed that age was the strongest independent predictor of elevated SDF (t=4.8993, P<0.0001), followed by abstinence (t=3.7950, P = 0.0002) and sORP (t=3.1856, P = 0.0015). However, if the percentage of DNA-fragmented sperm was used as outcome variable, sORP was the strongest independent variable (t=6.9800; P<0.0001), followed by age (t=6.4633; P<0.0001) and abstinence (t=2.8695; P = 0.0042). If the total number of DNA-damaged sperm was used as dependent variable ejaculatory abstinence contributed most to a high number of DNA-damaged sperm, whereas age was not included in the statistical model. For sORP, abstinence was the strongest predictor followed by age. The variance inflation factors (VIF) close to 1 indicate no or neglectable multicollinearity (**Table 6**).

**Table 6:** Multiple linear regression analysis for the investigation of the contribution of sexual abstinence, age, sORP, sORP/vitality, and sperm vitality to SDF.

Dependent variable: SDF ≤20% or >20% (yes-no)								
Independent variables	Coefficient	Std. Error	95% CI	t	P	r_partial_	r_semipartial_	VIF
(Constant)	-0.0715	0.0965	-0.2611 to 0.1180	-0.7408	0.4591			
Abstinence (d)	0.0418	0.0110	0.0202 to 0.0635	3.7950	0.0002	0.1366	0.1334	1.019
Age (yrs)	0.0110	0.0022	0.0066 to 0.0154	4.8993	<0.0001	0.1752	0.1722	1.006
sORP (mV)	0.0016	0.0005	0.0006 to 0.0026	3.1856	0.0015	0.1149	0.1120	1.018

Dependent variable: Percentage of DNA-fragmented sperm (%)								
Independent variables	Coefficient	Std. Error	95% CI	t	P	r_partial_	r_semipartial_	VIF
(Constant)	-2.2836	3.6016	-9.3540 to 4.7867	-0.6341	0.5262			
Abstinence (d)	1.1783	0.4106	0.3722 to 1.9844	2.8695	0.0042	0.1037	0.0976	1.019
Age (yrs)	0.5395	0.0835	0.3756 to 0.7034	6.4633	<0.0001	0.2285	0.2197	1.006
sORP (mV)	0.1317	0.0189	0.0947 to 0.1687	6.9800	<0.0001	0.2457	0.2373	1.018

Dependent variable: Total Number of DNA-damaged sperm (10^6^)								
Independent variables	Coefficient	Std. Error	95% CI	t	P	r_partial_	r_semipartial_	VIF
(Constant)	-3.3963	3.9438	-11.1384 to 4.3458	-0.8612	0.3894			
Abstinence (d)	5.8954	0.8405	4.253 to 7.5455	7.0138	<0.0001	0.2469	0.2431	1.015
sORP (mV)	0.1606	0.0386	0.0848 to 0.2365	4.1572	<0.0001	0.1493	0.1441	1.015
Age (yrs)	Not included in the model							

Dependent variable: sORP (mV)								
Independent variables	Coefficient	Std. Error	95% CI	t	P	r_partial_	r_semipartial_	VIF
(Constant)	40.1161	3.7071	32.8471 to 47.3851	10.8214	<0.0001			
Abstinence (d)	2.2421	0.4321	1.3948 to 3.0894	5.1888	<0.0001	0.0980	0.0979	1.000
Age (yrs)	0.2214	0.0910	0.0430 to 0.3998	2.4339	0.0150	0.0461	0.0459	1.000

By using the option of ‘backward’ entering of variables into the model (first enter all variables into the model and next remove the non-significant variables sequentially), the variable sORP/vitality was removed from the regression equation.

## Discussion

The standard semen analysis according to WHO guidelines is considered a cornerstone in the assessment of the infertile male. It is a high complexity medical analysis comprising of important parameters such as sperm concentration, motility, and normal sperm morphology (14). As an extension, advanced testing for SDF and seminal oxidative stress have repeatedly been suggested in recent years (36–39). Although repeated studies, including meta-analyses, have shown SDF and oxidative stress to be predictive of fertilization, embryo development and pregnancy (40–42), the benefits of SDF and oxidative stress testing for the clinical management and reproductive outcome are still a matter of debate as these tests are viewed not essential for clinical decision-making (32, 33, 43). This might have various reasons including the lack of generally accepted methodologies with validated clinical thresholds or high inter-assay variability.

With regard to the association of the standard semen parameters with the duration of ejaculatory abstinence, our data confirm the general trend of higher values for sperm concentration and semen volume (21, 22, 44) as well as lower values for motility and viability with increasing times of sexual abstinence (23, 45, 46). Several studies have shown that extended periods of ejaculatory abstinence are associated with elevated levels of seminal oxidative stress and SDF (21, 25, 47). Results presented in this study confirm the relationships between the SDF and oxidative stress as well as the duration of ejaculatory abstinence and are consistent with the processes of sperm production, maturation, transport and storage. During extended periods of ejaculatory abstinence, sperm are stored in the epididymis, hence are exposed to reactive oxygen species (ROS) for a longer period of time (48, 49). Due to their limited intracellular repair mechanisms and extraordinary high content of polyunsaturated fatty acids in the plasma membrane (50–52), sperm are highly prone to oxidative assaults resulting in lipid peroxidation and DNA damage caused by malondialdehyde or 4-hydroxy-2-alkenals, which are highly mutagenic and genotoxic (53–55).

The WHO recommends a period of ejaculatory abstinence of 2 to 7 days prior to semen production (9), a period that has repeatedly been questioned. ESHRE suggests a much narrower range of 3 to 4 days of abstinence (24, 56). The reason for these different ranges might be because of different purposes. While the WHO manual is rather focussed on reliable diagnostic semen parameters such as sperm count or motility, the ESHRE recommendation is focused on the time window for optimum sperm functionality for *in vitro* fertilization (57, 58) which, as shown in this study, includes sperm DNA fragmentation.

The lack of standardized periods of sexual abstinence has direct implications for practitioners ordering an SDF test or preparing the patient for assisted reproduction. Using ROC curve analysis, this study clearly shows that abstinence periods should not exceed 4 days as the average SDF value and the prevalence of patients presenting with high SDF increased significantly after longer abstinence periods. Therefore, considering the importance of the contribution of intact DNA for a successful reproductive outcome, the period of ejaculatory abstinence should be taken into account when requesting a diagnostic SDF test. The association of shorter abstinence times, even as short as 2 hours, with reproductive outcome parameters such as fertilization, embryo development, implantation and pregnancy together with lower SDF rates has been shown (59–61).

Research showed that SDF and oxidative stress are also positively associated with the patients’ age (62–64), a result which, in this study, could only be confirmed for SDF as we did not observe any association between age and sORP and normalized ORP. Yet, considering that SDF and oxidative stress are (i) significantly associated with fertilization, embryo development and pregnancy (41, 65–68), (ii) age (63, 64, 69), and (iii) the duration of ejaculatory abstinence (25, 70, 71), the period of abstinence will have a significant effect on the use of SDF and oxidative stress as diagnostic tests and may thus influence the clinical decision making if health practitioners have to rely on the outcome of the test. Therefore, it is important to investigate the impact of age and ejaculatory abstinence as factors that can influence the outcome of oxidative stress and SDF determinations.

The general associations of SDF and oxidative stress with the duration of ejaculatory abstinence have been confirmed in this study. However, we went further and, based on the frequently used cut-off value of 20% DNA-damaged sperm in the HaloSperm test (31, 32), calculated thresholds for abstinence (>4 days), age (>37 years) and sORP (>59.9 mV). Although the calculated predictive power is only relatively weak, the prediction is highly significant which is likely due to the multifactorial causation of SDF. For the parameters investigated in this study, namely abstinence, age and sORP, multiple linear regression analysis has shown age and ejaculatory abstinence to be significant independent predictors of elevated SDF values not only of high SDF but also of seminal oxidative stress.

Seminal oxidative stress measured as sORP, the patients’ age and ejaculatory abstinence had significant influence on high SDF. This study also revealed an almost 3-times higher probability of elevated SDF values in patients older than 37 years with seminal oxidative stress. For these patients it would make sense to recommend a shorter period of abstinence and/or the supplementation with antioxidants if no other cause for the high SDF such as a genital tract infection or varicocele has been detected. Therefore, the patient’s age, oxidative stress and abstinence as contributing factors to high SDF have to be considered when interpreting the results of SDF and oxidative stress testing as well as the management of the patient. Alternatively, one could think of different optimal abstinence times for different parameters and different age groups as suggested by Chen et al. (72).

While definition of a clear upper abstinence threshold for the determination of SDF is certainly beneficial for the diagnostic and management of a patient, this study also has limitations. Since the patients were instructed to follow WHO guidelines with a period of sexual abstinence between 2 and 7 days, we only have 6 patients with a longer abstinence period in our patient cohort. Therefore, it was not possible to provide a more detailed picture of the levels of SDF and oxidative stress after longer abstinence periods. However, since both SDF and sORP significantly increased even earlier than 7 days, it is unlikely that these parameters will improve at longer periods of abstinence. Moreover, since only viable sperm are able to reach and fertilize an oocyte, and sperm viability is significantly correlated with SDF (73), the determination of SDF in viable sperm might increase accuracy and predictive value of the test as proposed by Muratori et al. (74). Another limitation is that this study is a retrospective study which did not include other factors that can cause oxidative stress such as poor lifestyle, smoking, obesity or diabetes and therefore influence SDF.

## Conclusions

In conclusion, the multiple linear regression analysis in this study clearly showed that SDF is dependent on the duration of abstinence, the patients’ age and seminal oxidative stress with the dominance of the independent variable being dependent on which parameter was chosen as dependent variables, high/low SDF, percentage of DNA-fragmented sperm, or the total number of DNA-fragmented sperm. The ROC curve analysis and the analysis of the stratification of the abstinence for elevated SDF (>20%) resulted in a threshold for abstinence of a maximum of 4 days of abstinence. Considering that SDF is significantly influenced by seminal oxidative stress, patient’s age and the duration of abstinence, the study also clearly shows that these contributing factors have to be considered when requesting and interpreting an SDF test. It will also have direct impact on the management of the patients. Nevertheless, more detailed prospective studies are necessary to discern the benefits of considering ejaculatory abstinence when testing for SDF. This might be particularly important for specific patient groups such as oligozoospermic or asthenozoospermic patients.

## Data Availability

The datasets presented in this article are not readily available because According to institutional policy clinical data cannot be shared. Requests to access the datasets should be directed to ralf.henkel@logixxfertility.com.
